# An Overview of Non-Destructive Testing Methods for Integrated Circuit Packaging Inspection

**DOI:** 10.3390/s18071981

**Published:** 2018-06-21

**Authors:** Pouria Aryan, Santhakumar Sampath, Hoon Sohn

**Affiliations:** Department of Civil and Environmental Engineering, KAIST, 291 Daehakro, Yuseong-gu, Daejeon 34141, Korea; pouria.aryan@kaist.ac.kr (P.A.); santha@kaist.ac.kr (S.S.)

**Keywords:** IC packaging, defect detection, non-destructive testing, X-ray, scanning acoustic microscopy, surface acoustic waves, thermography, ultrafast laser

## Abstract

The article provides a review of the state-of-art non-destructive testing (NDT) methods used for evaluation of integrated circuit (IC) packaging. The review identifies various types of the defects and the capabilities of most common NDT methods employed for defect detection. The main aim of this paper is to provide a detailed review on the common NDT methods for IC packaging addressing their principles of operation, advantages, limitations and suggestions for improvement. The current methods such as, X-ray, scanning acoustic microscopy (SAM), infrared thermography (IRT), magnetic current imaging (MCI) and surface acoustic waves (SAW) are explicitly reviewed. The uniqueness of the paper lies in comprehensive comparison of the current NDT methods, recommendations for the improvements, and introduction of new candidate NDT technologies, which can be adopted for IC packaging.

## 1. Introduction

As the sizes of electronic circuits are miniaturized from µm to nm, the speed and capacity of integrated circuit (IC) packaging has been improved dramatically [1]. The demand towards smaller size in the packaging has made the IC packaging, one of the fastest-growing technologies over the past half century [2]. As the sizes are getting smaller, the IC packaging are getting more complex, more challenging manufacturing processes are developed and more faulty and damaged packaging are produced. Based on the current trend, there is a growing demand for more advanced, reliable and robust inspection and testing methods. 

Non-destructive testing (NDT) has played a crucial role in the integrated circuit (IC) packaging industry, and the relevant research has been conducted intensively since the early1970s [3]. Generally the NDT methods for integrated circuit packaging inspection can be divided in three major categories:

(1) Functional inspection (electrical tests to find functional faults);
(2) External inspection of the structure of the package;
(3) Internal inspection of the structure of the package.

The conventional inspection methods for the IC packaging have been utilised to assess the condition of the IC components for more than half century, but the non-destructive evaluation process recently has become more challenging due to complex structures of the packaging and related defect types. Alongside the visual inspection, X-ray [4,5], Scanning Acoustic Microscopy (SAM) [6,7] can be named as the oldest methods used to inspect the electronic packaging since early 1970s [3]. Over the past two decades, a wide variety of other NDT methods have been adopted for testing and inspection of IC packaging including; infrared thermography (IRT) [8,9] and surface acoustic waves (SAW) [10,11,12,13]. 

This paper aims to provide an overview on the past and current non-destructive testing methods dealing with the structural integrity of IC packaging (categories 2 and 3). In Section 2, an introduction to the evolution of IC packaging, the related defect types, the current packaging technology and associated challenges are described. Section 3 reviews the common NDT methods used for IC packaging with detailed discussions on their respective advantages, disadvantages, limitations and principles of operation. Section 3 ends introducing an advanced technology candidate to be utilized for IC packaging inspection. Section 4 provides a table that compares various technical aspects of the current NDT methods followed by discussions on the future direction and recommendations on the improvement of NDT methods and in-line application. The paper ends with conclusions in Section 5. 

## 2. The Evolution of IC Packaging, Related Defects and Current Challenges for IC Package Inspection

The evolution trend of microelectronic packaging (or IC packaging) since 1970 can be described as dual in-line package (DIP) and quad flat package (QFP) back in the 1970s, pin grid array (PGA) in 1980s, ball grid array (BGA) in 1990s, and flip chip ball grid array from 2000s. DIP packaging was initially invented in the early 1960s to compensate the need for more signal supply and connectives in integrated circuits. Lifted or missing leads and solder bumps are the most significant defect types for DIP packaging [14]. In 1975, another type of electronic packaging is introduced—quad flat package or QFP. The structure of the QFP is very similar to DIP, but the main difference is that the leads have been extended from all four sides of the surface mounted IC component. In terms of the inspection of the packaging, the change put a higher demand on the accuracy of the soldering process and more importantly the alignments of the packaging [15,16]. In 1985, pin grid array (PGA) was designed. A similar concept to increase the number of connectives for PGA and BGA was utilized. The new design allowed dramatic increase in the number of pin counts with the same dimensions [16]. Defect types in DIP, QFP and PGA packaging are very similar. Solder defects, lifted lead, missing lead can be listed as critical defects in the packaging. 

For the inspection of DIP, QFP and PGA packaging, usually X-ray or automated optical inspection (AOI) are utilised to measure height and volume of solder leads. AOI autonomously scans the device under test using a camera mainly looking for soldering defects [17]. Followed by PGA in 1990, ball grid array (BGA) is introduced to the industry. BGA packages are adopted for the devices such as microprocessors. The main advantage of BGA over PGA is that the whole bottom surface of the package is used for connectors, and, due to the shorter leads, they have higher speed performance. On the other hand, soldering process of BGA packaging requires more precise quality control [18]. Soldering fault is one of the most significant defects for BGA packaging. X-ray, thermography and SAM have been adopted in various research studies and industrial applications for BGA packaging [4,5,8,18,19]. 

**Figure 1:**
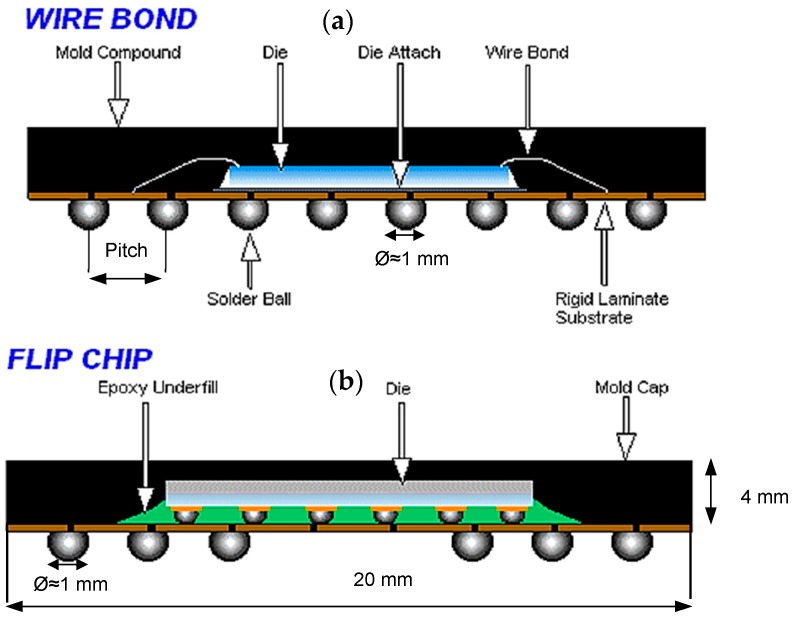
Comparison of wire bond BGA and FCBGA packages: (**a**) Wire bond BGA: the die faces up and attached to the package via wires, (**b**) FCBGA: the die faces down and attached via solder bumps (courtesy of Amkor Technology, Inc., Tempe, AZ, USA).

With continuous and increasing demand on performance and capacity of the IC packaging, flip chip ball grid array (FCBGA or flip chip) was produced in 2000 and soon became one of the most common and suitable types of IC packaging in the industry [6,7,20]. Flexibility in design, higher signal density with much smaller size are some of the advantages of the FCBGA packaging. FCBGA packages can be mounted on standard printed circuit boards [14]. Figure 1 schematically compares the wire bond BGA and FCBGA packaging with different parts marked.

Defects more frequently occur in FCBGA in comparison with the other types of packaging, and the problem associated with defects is mainly due to the new materials such as lead-free solder bumps or low-k underfill (small dielectric constant) used in the structure of FCBGA [21,22]. Defects in FCBGA occur in different parts of the packaging. In solder joints; void, crack and missing soldering bump are more common amongst the others [21]. In other parts of the packaging such as underfill, delamination, crack or void are the critical defect types [20]. 

**Figure 2:**
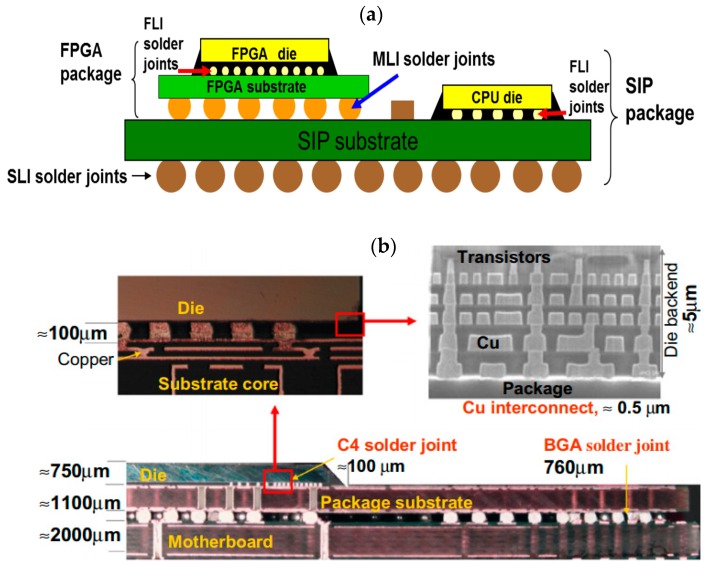
3D packages with multi-layer components (**a**) SIP package with POP configuration [25] and (**b**) Cross sections of a 3D package with stacked die and TSV configuration. Approximate dimensions and zoomed parts which marked with squares [26].

The market demands for even smaller size, ultra-light and ultra-thin packaging resulted in 3D packaging in early 2000s [23]. The main aim of 3D IC packaging is to achieve better performance with reduced power, size, and cost at the same time [24]. Typical Si chips in 3D packages are about 50–100 μm thick, and 90% thinner than the previous packages [23]. Two common configuration for 3D packages are package on package (POP) or stacked die with though-silicon via (TSV) which are presented in Figure 2 with approximate dimensions and cross sections of the layers. In comparison with packages with POP configurations, stacked die with TSV have much smaller solder bumps and more complex interconnections which create more challenges for defect detection methods.

Due to multi-layer nature of the 3D packaging and dramatic increase in number of the components in the package (stacking of multiple chips), the inspection of 3D packaging faces with very serious challenges. The challenges can be listed as: (1) complexity to differentiate the defect between different layers such as stacked dies, SIP substrate or interconnections. (2) The potential to have new defects due to vertical interconnections and complex alignments of the layers. (3) Accessibility to the chip becomes much more limited and complicated comparing to conventional packages. (4) New features such as µ-bumps and thinned stacked dies add more defect types and (5) in terms of chip package interaction (CPI), thermal and mechanical issues become more significant.

Most common defect types of 3D packaging are delamination, crack, misalignment and void in µ-bumps, crack in substrate, delamination and void in under fill or void and delamination in through-silicon via (TSV) [25,26]. For the inspection of microelectronic packaging, the current solution is usually X-ray, lock-in thermography or AOI methods.

AOI uses visible light and is capable of obtaining multiple images from different lighting angels in one scanning operation the current resolution of AOI is approximately 1 µm and it takes several seconds to perform the scan. Automated X-ray inspection (AXI) operates same as AOI, except that the method uses X-ray instead of visible light to inspect internal components of microelectronic packaging. AOI method usually is adopted to inspect the external integrity of the structure of the package and the connections while AXI can be utilised for inspection of internal and external integrity of the packaging simultaneously. 

## 3. NDT Methods for IC Packaging

**Figure 3:**
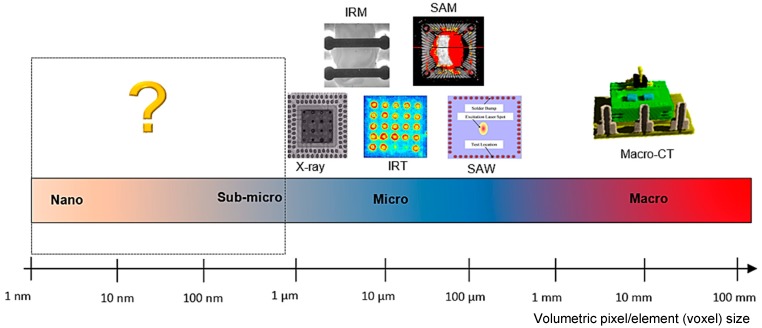
Schematic demonstration of the technology gap between the IC package size and the spatial resolution of NDT methods.

According to IPC (the association connecting electronics industries) Roadmap for Interconnect Technology [27], simultaneous size reduction and performance improvement still remain as the main challenges of IC packaging [28]. It has been predicted that the structural size of IC packaging will be in the order of few nanometers in next several years [28]. Considering the aforementioned trend, the gap between the IC package size and the spatial resolution of the inspection methods is shown in Figure 3 in terms of volumetric pixel/element (voxel) size of the packaging. 

Based on the current gap, the main limitations of the conventional NDT methods are: (1) lack of spatial resolution to meet the requirements of the industry (sub µm scales), (2) lack of automation to assess the integrity of the component, (3) long inspection time not suitable for in-line inspection, and (4) limited inspection capability for inner components of the IC packaging [29,30]. 

### 3.1. X-ray

When X-ray is transmitted from an X-ray source to a receiver, X-ray photons interact with the specimen and a portion of X-ray photons are absorbed by the specimen. The level of X-ray absorption depends on the elemental composition of the material and geometries of the specimen. Therefore, presence of defects causes variation to the level of the absorbed energy or the transmitted energy at the receiver. Based on the aforementioned characteristics, the density and shape characteristics of various objects can be measured and inspected by X-ray [31]. A typical X-ray inspection system consists of three components: X-ray source (tube), X-ray detector and a fixture to hold and control the position of the sample for inspection. For the source, microfocus tubes are utilised providing spatial resolution down to 1 µm. The resolution depends on the size of the focal spot (or focus), the area within the tube head that emits the X-ray beam. The most common type of a detector is a combination of video camera and image intensifier that converts X-rays into visible light. 

**Figure 4:**
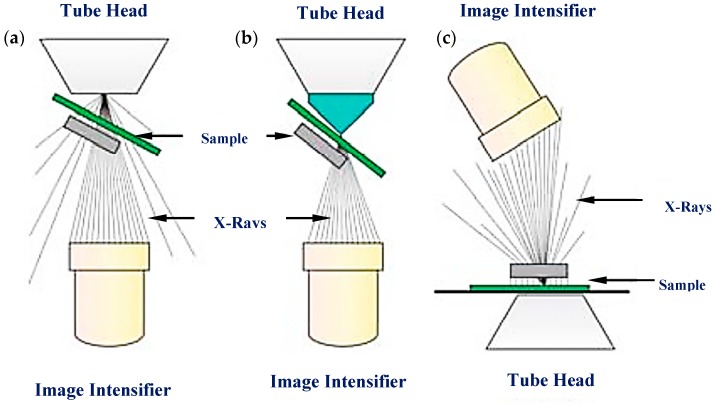
Different configurations of the isocentric motion technology X-ray inspection method to achieve oblique viewing (3D): (**a**) conventional, (**b**) advanced, and (**c**) close-to-focus [32].

X-ray inspection methods are considered some of the oldest methods in IC packaging inspection [3]. Previously, conventional 2D X-ray inspection methods have been adopted to inspect various parts of IC packaging due to their ability to penetrate substrate materials and detect hidden faults such as voids, delaminations and cracks, within the order of mm, for electronic packaging by providing top-down views of the samples [32]. One major disadvantage of the conventional X-ray methods is that the X-ray resolution cannot provide accurate assessment of BGA and flip-chip components within the range of ten µm [33]. In addition, top-down views are usually insufficient for a thorough assessment of the solder connections in recent electronic devices. To tackle the limitation, isocentric motion technology (IMT) technology was developed [32]. The IMT X-ray inspection method has a mechanism that tilts and rotates the sample under inspection within the X-ray beam cone, creating an oblique view. The oblique viewing provides the third dimension information about solder connections and etc. within the devices. Figure 4 shows the schematic configurations of the IMT X-ray method.

**Figure 5:**
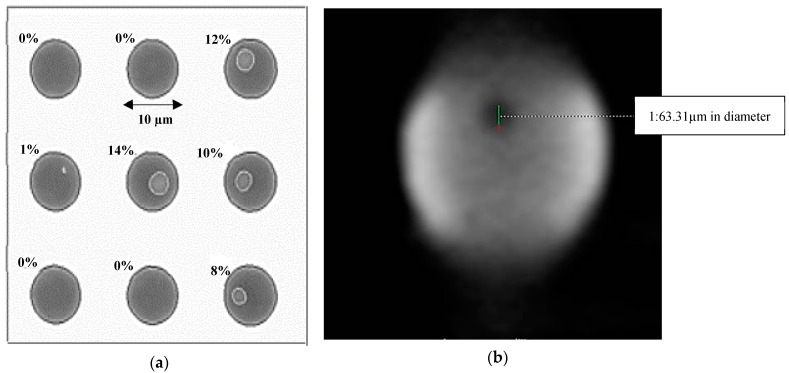
(**a**) Detection of a wafer bump void based on the ratio of the void to the total area [32], (**b**) Void detection in µm level using metallographic microscopic image [36].

**Figure 6:**
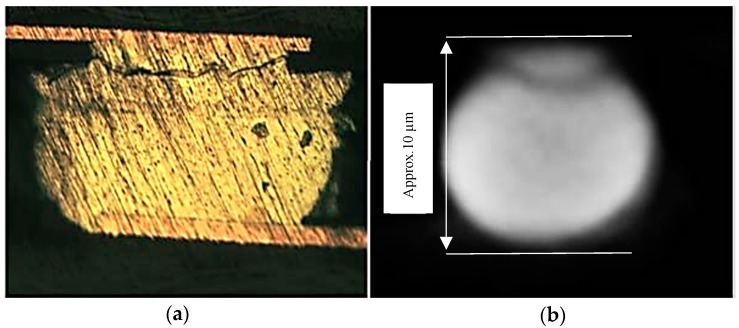
Crack detection in a micro solder bump: (**a**) metallographic microscopic image; (**b**) slice-by-slice inspection of μCT images [36].

To meet the industry demands for higher accuracy and resolution, improved X-ray methods have been developed [34]: X-ray laminography [4], microlaminography [18] and microscopy [35,36]. According to Sassov et al. [18] … “combination of classical approaches with modern digital acquisition techniques allows improving spatial resolution to µm”. For instance, digital algorithms have been developed to reconstruct individual layers from a set of laminography and microlaminography X-ray measurements. In X-ray laminography and microlaminography, it is possible to analyze distinct layers of flat objects with specific depth resolution of micron [18]. The improved X-ray methods have successfully detected defects such as wafer bump voids with about 2 µm in diameter (Figure 5) [32,37] and cracks in solder interconnects (with size of less than 10 µm) [36] (Figure 6). In addition, the improve X-ray has been used successfully to inspect BGA joints [4,5], solder joints [31], flip chip joints [18,35,38], and FBG solder joints [35].

Although the improved X-ray inspection methods have been confirmed promising to inspect micro bump soldering and alignments of the various parts of packaging (under 10 µm in size), the problem associated with resolution still remains as a challenge for detecting submicron defect. Furthermore, long data acquisition and processing time (few hours) and radiation issue are other drawbacks of the current X-ray methods [39]. The ongoing research is more focused on 3D X-ray imaging using Computed X-ray tomography (CT), which provides three dimensional images of complex structures such as 3D IC packaging. Currently inspection of a SIP package using 3D CT takes from 3 min to 10 min depending on the resolution and package size. 3D CT is able to inspect different layers of 3D packaging provided that the image reconstruction process is complete. Recent advancement in computer technology has made CT a powerful tool for NDT applications and further developments are in progress to shorten the data collection time as well as to improve the resolution. The future development of X-ray method should address multifunctional X-ray tubes, more advance X-ray intensity control to achieve better performance and image quality, high speed digital detectors for a much faster image recording and reconstruction.

### 3.2. Scanning Acoustic Microscopy (SAM)

**Figure 7:**
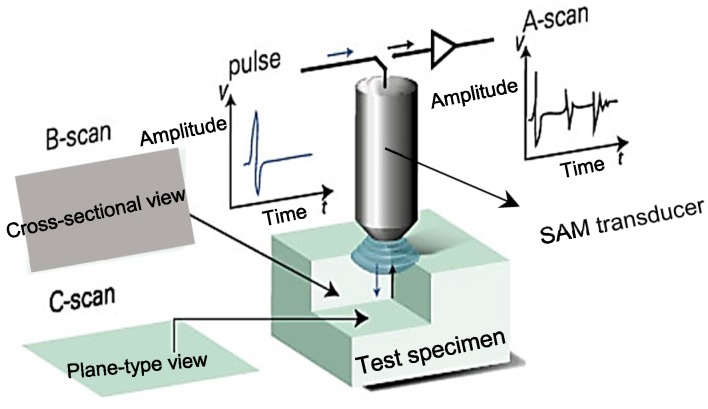
Schematic picture of three scan modes of SAM method (Courtesy of PVA TePla^®^ Company, Wettenberg, Germany).

SAM method uses acoustic waves as a source to create visual images of variations in the mechanical properties of samples. The main part of the acoustic microscope is the probe or transducer (usually a piezoelectric or loudspeaker depending on the applications), which converts electrical signals into acoustic signals. The acoustic waves are focused and transmitted to the sample through a couplant (normally water). When the generated waves interact with the sample, part of the waves reflects back to the transducer and the other part is transmitted. Subsurface imaging is also possible due to the fact that acoustic waves can penetrate opaque solids. As a result of the interaction of sound with samples. SAM is capable to estimate properties such as thickness, stiffness, density, shape, roughness and attenuation [40]. Generally, SAM provides three scan modes; A-, B-, and C-scan. A-scan displays a reflection signal measured at a single measurement point in a pulse-echo mode. The height of the vertical spikes corresponds to the strength of the echo from the specimen, and the position of the vertical spikes along the horizontal time axis represents the wave travel time in the depth direction [41]. By measuring the arrival time of the peaks, the thickness of the specimen (or the location of internal defect) can be estimated. The principle of B-Scan is the same as that of A-Scan except that the transducer in B-Scan is scanned over a line within the specimen rather than kept in a fixed position [40]. From the B-scan, the cross-sectional image along the scanned line can be obtained visualizing internal features such as defect and its distance from the scanned surface. With C-scan, plane views of the cross sections parallel to the surface of the specimen is possible [42]. Figure 7 shows the schematic of three SAM scanning modes.

**Figure 8:**
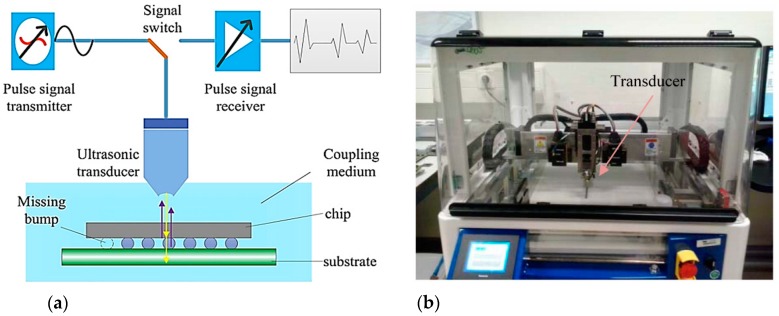
SAM method for inspection of IC packaging: (**a**) schematic diagram of SAM method [50], and (**b**) SAM inspection setup [19].

The utilization of the SAM method for failure analysis of the surface and internal microstructure of solid or IC packaging goes back more than two decades [6,7,43]. SAM method can detect flaws such as solder bumps defects, laminar cracks (with sizes less than 50 µm and frequency range of 100–150 MHz) [44], underfill defect such as crack and delamination (test chip with size of 5.6 × 6.4 mm^2^) [45], bump defects [46], void defects (as small as 125 µm in diameter) [47], and solder bridging (when two solder joints melted together, forming an unintended connection between the two) [48] and delamination at the IC (with approximate size of 100 µm). The frequency of the ultrasonic signals generated for IC package inspection is typically within 15 up to 300 MHz [48]. Figure 8 provides schematic illustration of SAM method for inspection of IC packaging and the actual SAM setup available in TNO Eindhoven (The Netherlands). The frequency of the ultrasonic signals can be increased into GHz range, which makes it possible to detect defects even in the sub-micron-range [49]. 

**Figure 9:**
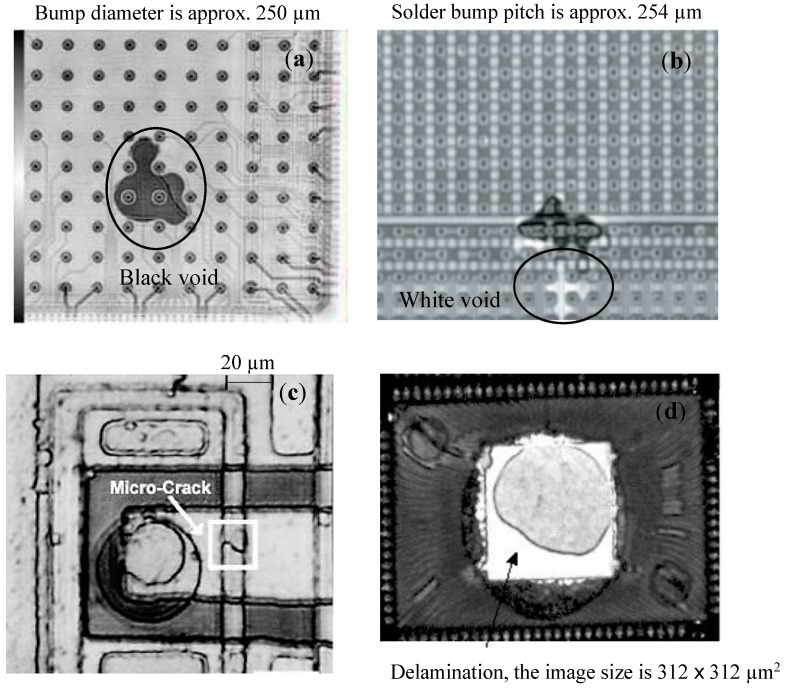
Various types of defects detected by SAM: (**a**) Black void below underfill level, (**b**) White void above underfill [19], (**c**) Micro-crack at wafer level, and (**d**) Delamination between the die top and the encapsulates [49].

The resolution of microscopic image depends on the acoustic frequency, the material properties and aperture of the transducer [51]. Figure 9 shows samples of different defects successfully detected by SAM. 

The SAM method is an ideal method to study the thickness of layers and delamination inside material, underfill delamination and voids defects. However, defects located on edges of packaging cannot be detected using SAM, due to so called the edge effect (distortion of the reflections from the edge) [52]. Other limitations of SAM are the requirement for a coupling medium (usually deionized water) to propagate acoustic energy from a transducer to the specimen and lack of resolution in sub µm levels [53]. Although the resolution can be improved at the expense of the decreased penetration depth by driving the transducer at higher frequencies in GHz range. Consequently, there is always a compromise between the resolution and the penetration depth. There is an ongoing research to combine SAM and wave propagation modelling to better understand the trade-off between the resolution and the penetration depth [54,55].

### 3.3. Surface Acoustic Waves (SAW)

Surface acoustic waves (SAWs) are traveling waves along the surface of a material, the amplitude of which normally decays with depth into the substrate. SAW in general, can be generated and measured by either contact transducers (such as piezo materials) [56,57], contact actuator and noncontact sensor [58,59,60] and fully noncontact systems such as laser-based [53,61] or based on air-coupled transducers [62]. 

**Figure 10:**
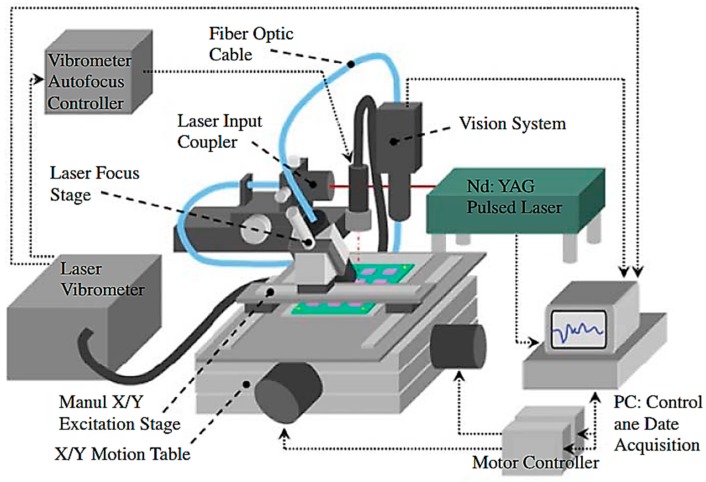
Schematic picture of a SAW inspection system for IC packaging (noncontact laser–based) [65].

History of utilizing SAW methods for electronic package inspection goes back to 1980s [63]. Most of SAW methods are using noncontact laser excitation and sensing [64]. The main components of the laser-based systems are Nd YAG pulsed laser, control unit, data acquisition system, laser vibrometer (Figure 10). A pulsed laser as an excitation source induces heat (in thermo-elasto regime) in the electronic packages results in generation of ultrasound waves, and a laser Doppler vibrometer measures the transient out-of-plane displacement response. Through the response measurement over the specimen surface, the system identifies defect such as missing solder bump based on different responses [65].

Several post-processing analyses for the SAW inspection system have been reported in the literature to identify the variation of the surface wave responses produced by defect [65]. The post-processing analyses include correlation coefficient analysis [66] in the time domain, spectral analysis [29] in the frequency domain, pattern recognition [67], wavelet analysis [68], and local temporal coherence analysis [69]. 

Noncontact SAW methods have been used to identify defects such as cracks (normally larger than 15 µm) and voids in microsolder bumps (approx. 200 μm in diameter and spaced with a pitch of 450 μm) [69,70,71,72,73,74,75,76,77], underfill [13] in flip chip [53,64,68,78], PBGA [77] and BGA [79,80,81]. The range of inspected chip sizes is reported within 10 mm in length and width and 0.5 mm in thickness. The sensor head has a spot size of 3 µm, and the vibrometer has a displacement resolution of 0.1 nm and a bandwidth from 25 kHz to 20 MHz.

**Figure 11:**
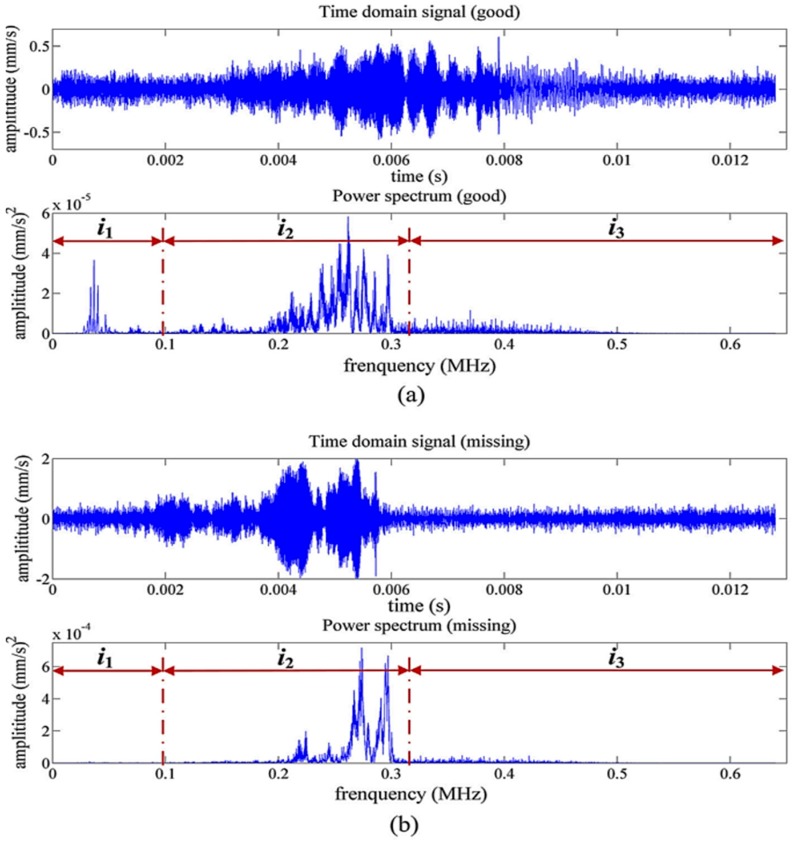
Representative SAW responses in the time and frequency domains obtained from (**a**) an intact flip chip and (**b**) a flip chip with a missing solder bump [82].

Representative time signals and power spectrums of an intact flip chip and a flip chip with a missing solder bump are shown in Figure 11. The power spectrum signals are presented in the frequency domain and divided in three regions (i_1_, i_2_ and i_3_). Comparison of Figure 11a,b reveals that the missing solder bump resulted in the missing power spectrum peaks within i_1_, and i_2_.

**Figure 12:**
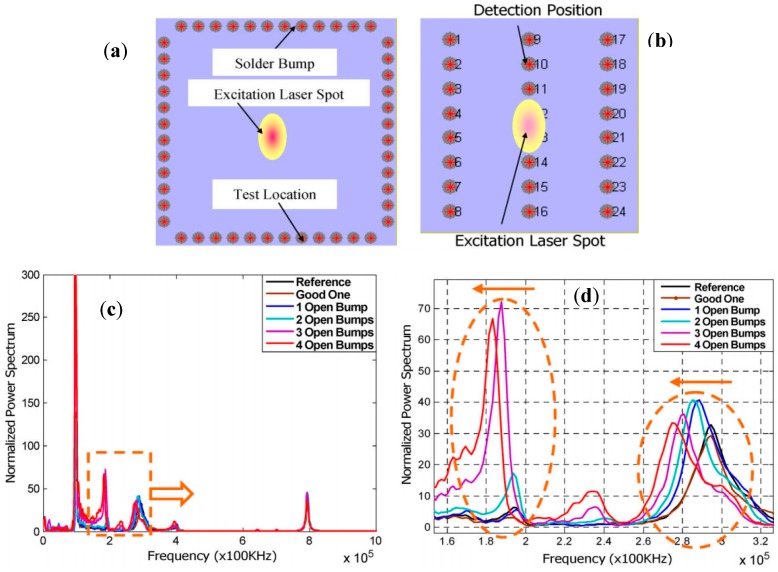
Flip chip solder bump inspection using SAW: locations of laser excitation and sensing for (**a**) Test specimen I and (**b**) Test specimen II (with different solder bump arrangements), (**c**) Power spectrum of SAW under pulsed laser excitation, and (**d**) Close-up of the power spectrum in range of 160–320 KHz for test specimen II [68].

Figure 12 shows the locations of laser excitation and sensing on two different flip chip samples with the corresponding frequency spectrum of measured SAW for solder bump inspection. The waveforms from two healthy chips match very well, one of which is selected as the reference. However, the signals from the chips with open solder bumps differ from the reference one. The more open bumps a chip has, the larger difference is shown between the responses of the faulty chip and [68].

The SAW method is capable of revealing the presence of voids, with an implied lateral spatial resolution about 100 µm. The method is mostly sensitive to voids and cracks. However, SAW method cannot access certain parts of the electronic package such as underfill and bump pitch smaller than 100 µm. Long inspection time is also another limitation of the method. 

### 3.4. Infrared Thermography (IRT)

Infrared thermography (IRT) is one of the most common NDT methods for material evaluation [83,84]. The basic working principle of IRT method is to measure the heat luminance from the surface within the electromagnetic spectrum region corresponding to the infrared (IR) wavelength (2–14 µm) and to record the temperature distribution of the surface (Temperature resolution can range from 0.020 °C to 0.075 °C, depending on the type of IR detector) [85]. IR detectors work in different infrared bands short, middle, and long wave bands; the mainly used are the middle wave (2–5 μm) and the long wave (8–12 μm) [86,87].

Utilization of thermography method for inspection of IC packaging goes back to more than half a century ago [88,89,90,91], and since then it has been one of the most promising NDT methods to inspect electronic packaging [8,9,92]. IRT is used to inspect flip chips [93,94], solder joint defects [94,95], edge defects [33], misalignment of solder bumps [96], presence and location of microsolder bumps (as small as approximately 100 µm in diameter and 250 µm in pitch) [94,97,98], silicon crack [99], underfill void [99], delamination defects with minimum size about 2 µm [100], and subsurface defects (up to 4 mm in depth) [101].

Either active or passive thermography methods are available. For thermal imaging, the passive method relies on natural heat emitted from the structures or structural components in service without any additional external heat source and the infrared detectors senses thermal radiation [102]. On the other hand, the active method exerts an external heat to the parts to be inspected using optical [8], mechanical [84] or electromagnetic [95] sources. The external heat creates thermal waves on the surface of the specimen, and the thermal waves reflect back from a defect or anomaly within the object under inspection [103]. 

**Figure 13:**
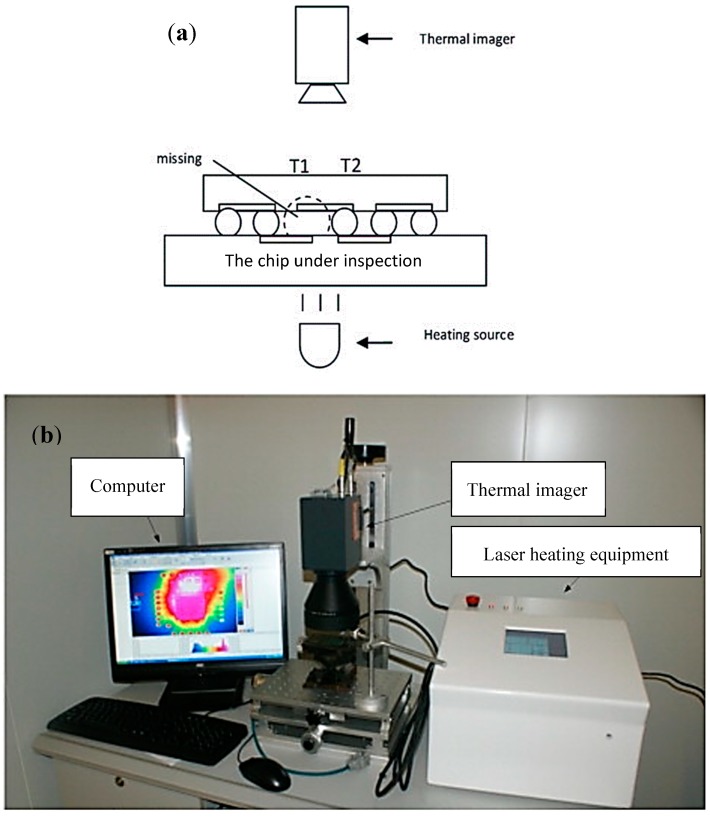
A represetative experimental setup for inspection of solder bumps using IRT method, (**a**) the inspection procedure, and (**b**) the different parts of the experimental setup [33].

The active thermography method has been utilised for delamination and moisture detection in composites [104], identification of buried mineshaft and canal seepage [105] and high density IC package inspection [9,93]. Pulsed and lock-in are the two major subcategories of the active thermography depending on the stimulation with the heat source. In the pulsed thermography, the sample surface is instantly heated using an optical flash. Over time the surface heat penetrates into the material, and subsurface defect changes heat-flow [94]. In the lock-in thermography, the sample surface is periodically heated by an input energy wave (i.e., thermal emitter, microwave or flash lamp) and thermograms are captured. When the input wave reaches areas within the object where the thermophysical properties are not homogeneous in relation to the surrounding material (i.e., at delaminations or inclusions), the input wave is partially reflected causing an interference pattern in the local surface temperature. The reflected wave oscillates at the same frequency as the thermal wave [106]. The active thermography methods have been adopted extensively to inspect the IC packaging (pulsed [94,95,107,108] and lock-in [92,93,101,109]). A sample setup (both schematic and actual) of the active thermography is shown in Figure 13. After the chip is heated by the heating source (laser, in this case), the missing solder bump in the chips influences the thermal conductivity process and therefore lead to different temperature distribution (T_1_ and T_2_ in Figure 13a). The IR camera is utilized to capture the temperature distribution. The infrared energy emitted by the chip and the surroundings is analyzed, post processed and visualized (Computer screen in Figure 13b). 

**Figure 14:**
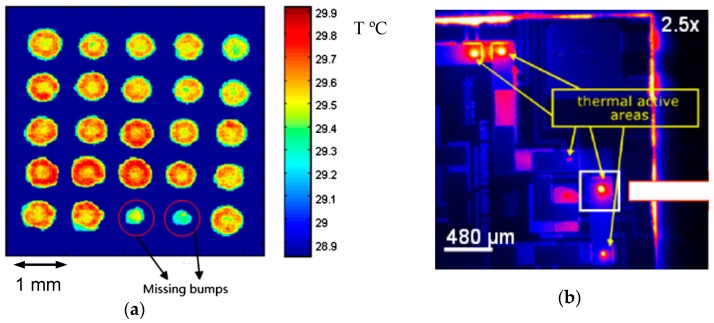
Defect detection using infrared thermography method: (**a**) missing bumps with lower temperature [33] and (**b**) failed device emitting increased heating [92].

A detailed technical review on the IR methods used for defect detection on electronics boards can be found in Hsieh et al. [86]. This review paper provides recommendations and essential consideration regarding the successful application of IR methods for IC packaging inspection and circuit card maintenance. Figure 14 shows two representative examples where the infrared thermography method is used for detection of missing bumps and failed devices with increased temperature. In Figure 14a, the thermal responses around the missing bumps are slightly lower than those of the surrounding intact bumps. On the other than, the failed device in Figure 14b emits significantly higher level of heating than surroundings.

In terms of 3D packaging, lock-in thermography has proven to be successful for defect detection specifically to locate short failures in stacked dies. Since heat propagation is time dependant lock-in thermography can define the depth and locations of hot spots induced by short failures in 3D packages. Although the infrared thermography method has shown very promising results for NDT applications, there are still challenging issues that need to be addressed: (1) The method can detect defects on the surface or only near the surface of the specimen. This is mainly because the penetration depth of heating is limited to the infrared wavelength. (2) Spatial resolution is limited to the pixel resolution of the infrared camera and the wavelength within the infrared range. To be more specific, because the wavelength of a middle range infrared camera is in the range of 3–5 µm, the best spatial resolution, which can be achieved with a close-up lens, is around 3.75 µm, dictating the smallest size of the detectable defect. Also in sub-micron orders, the temperature difference between the reflected heat waves is too small to differentiate the defect [33]. (3) The recorded results can be affected by surrounding temperature reflections and low contrast of infrared images [82]. (4) Heat propagation in active methods can potentially damage the specimen. Future development of the thermography can include combining analysis and decision making techniques such as pattern recognition or artifical neural network (ANN) to facilitate the assessment also using IR cameras with higher resolution can improve the range of operation for infrared thermography [110].

### 3.5. Other Methods

Additional methods used for IC packaging inspection include magnetic current imaging (MCI), infrared microscopy (IRM), time domain reflectometry (TDR) and so on. The magnetic current imaging (MCI) method operates based on measurements of the magnetic field associated with a flowing current. The method is able to map out hidden current-carrying wires by measuring the magnetic fields around them. The magnetic field images of the sample is converted into the current density images using Fourier transform inversion [111]. To locate the defect, the current density images are compared with defect–free samples. MCI method has been mostly successful for inspecting short circuit faults within electronic packaging. Although the resolution of micron is achievable, submicron spatial resolution is still a big challenge for the method [112,113]. 

Infrared Mmicroscopy (IRM) denotes a microscopy achieved at infrared wavelengths of 2 µm to 14 µm. For IC packaging, IRM method has been mostly used to inspect and locate underfill and cracks within the range of µm [100]. Unlike other optical microscopes with absorbent glass optics, an infrared microscope has reflective optics to allow the microscope to cover the entire spectral range of infrared light. For IC inspection applications and specifically for flip chip devices, the advantage of the IRM is that most silicon materials are transparent at wavelengths greater than 1 µm. This fact enables defects such as voids, delamination cracks and corrosion to be investigated while the chip is mounted on the substrate. 

The IRM provides device images with a spatial resolution of 2–3 µm. On the other hand, sample preparation is required (back surface of the sample should be polished to get good quality images). Also transparency of the sample under inspection is necessary, and the method is able to inspect samples only within a limited thickness range of up to 10 µm [114]. 

**Figure 15:**
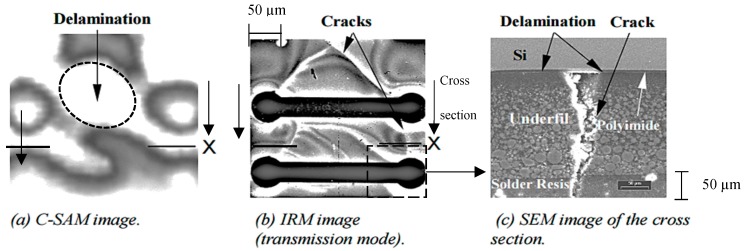
Representative images of defects in a sample specimen obtained by (**a**) C-SAM, (**b**) IRM, and (**c**) zoomed cross section of the specimen using SEM (zoomed area is marked on the IRM image) [100].

Figure 15 shows representative images taken by C-SAM, IRM and scanning electron microscope (SEM). C-SAM is not able to detect the crack whereas the IRM and SEM can. Here, a destructive testing with SEM method is performed for validation. 

**Figure 16:**
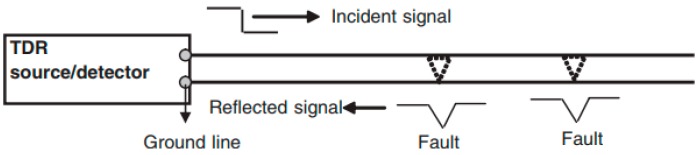
Schematic illustration of TDR defect detection method [115].

Time domain reflectometry (TDR) inspection method operates by sending an electrical pulse (usually a step or an impulse) and detecting reflections returning from impedance discontinuities along the controlled-impedance transmission path as shown in Figure 16 [115]. A defect alters the local impedance of the material, and the impedance variation produces reflections at the defect boundaries [116,117]. For instance, presence of a crack changes the amplitude of the reflected signal, and this amplitude change can be used as a signature for crack detection. TDR method can access to hard-to-access location, and the method has been used successfully to detect crack in flip chips [117,118]. In 3D packaging inspection, TDR can successfully detect defects in solder interconnects and open failures in general. TDR has been proven successful in SIP packaging with POP configuration. However, the application of TDR is limited only to conductive materials and baseline data is required to assess the presence of defect. 

### 3.6. Ultrafast Optical Laser Ultrasonics

With the current trend regarding package sizes mentioned in Section 2, the sizes of future IC packaging components will be in order of few nanometers. With denser configurations, there will be no direct access to components of the IC packaging. Considering the sizes and limited access to the components of interest, most of the current inspection methods often fall short. Consequently, future NDT methods should be able to offer noncontact inspection, better spatial resolution in submicron or nanometer order, and the potential to be part of in-line inspection systems with reasonable inspection time [119]. 

Amongst new advanced technologies, optical ultrafast lasers recently showed significant capabilities for NDT of IC packaging [120,121] such as resolution in sub-microns (nm), reasonable inspection time (within the order of couple of minutes) with a potential to be part of in-line inspection system, and ability to detect subsurface defects. The acoustic wavelength of the ultrafast optical lasers makes them an attractive candidate for the resolution gap mentioned in Figure 3. 

**Figure 17:**
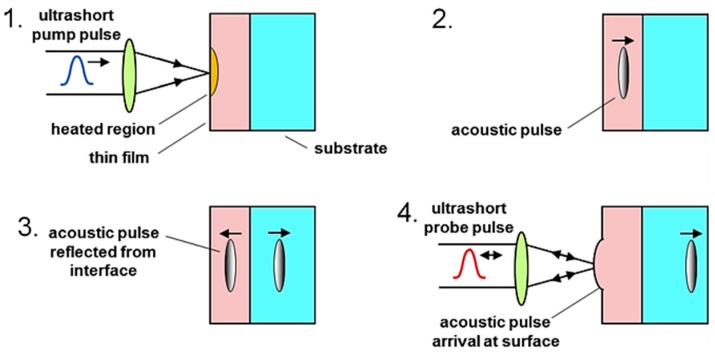
Elastic wave generation and sensing using ultrafast pump and probe laser pulses for detection of internal inhomogeneity (or the thickness of interface between thin film and substrate) [122].

Ultrafast lasers use pulse lasers with extremely short time intervals (typically <1 ps). Once such an optical pulse, known as pump pulse, hits on the surface of an opaque solid, a portion of the optical energy is absorbed by the specimen and converted to heat. The heat then leads to thermal stress that produces strain wave propagating in certain directions depending on the incident angle of the laser. A second ultrashort pulse called “probe” can detect the reflection of the acoustic pulse back to the surface. Reflectivity/phase change can be measured by adjusting the time delay between the pump and probe pulses in an optical delay line with no need for an ultrafast photodetector. An inhomogeneity (or the interface between different layers of material) beneath the surface, normally at nm to µm depths, can produce the reflection back to the surface as shown in Figure 17 [122]. 

**Figure 18:**
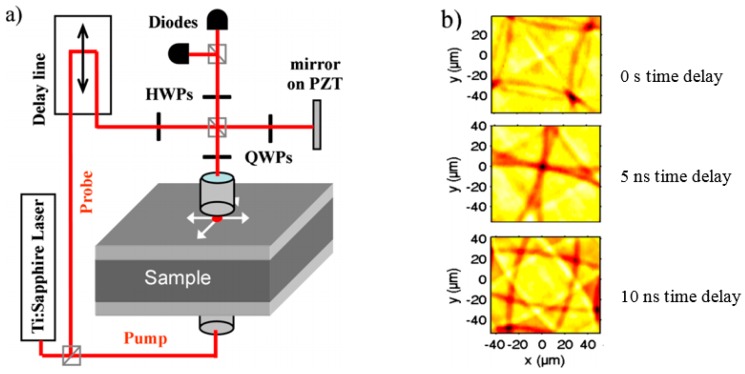
Imaging of a thin film sample using ultrafast optical laser ultrasonics: (**a**) experimental setup and (**b**) surface displacement measurements at three different pump-probe time delays. Changing the time delay between pump and probe (0 ns, 5 ns and 10 ns) provides set of measurements without ultrafast photodetector [123].

According to Zhang et al. [123], “when the optical spot size (typically a few microns) is much larger than the optical absorption depth (∼10–50 nm for example), the generated acoustic pulse can be accurately modelled as a superposition of longitudinal plane waves travelling normal to the surface”. Acoustic frequencies are usually in the range of 10–1000 GHz. Because of the short acoustic wavelength (within the nanometer range), ultrafast optical laser ultrasonic is a promising candidate to investigate thin films and nanostructures. Subsurface structures or inhomogeneous regions typically below nm to μm from the surface can reflect the acoustic pulse back to the surface. An example of the ultrafast imaging setup and snap shots of surface displacement imaging are shown in Figure 18. Pump and probe pulses are generated from a Ti:sapphire oscillator. The wavelength, repetition rate and duration of the pulse laser are 800 nm, 79.4 MHz and duration 0.1 ps, respectively. The incident pump laser is focused on one side of the sample, and the probe laser is sent through the interferometer. One half of the beam is focused on the sample and the other half is reflected on the mirror on a piezoactuator (PZT) to stabilize the interferometer. Photodiodes record the reflectivity changes due to surface displacements. Half-wave plate (HWP) and quarter-wave plate (QWP) are optical devices that alter the polarization state of the laser beams.

Picosecond and femtosecond lasers have been adopted successfully for material characterization such as exploring acoustic features on the nanoscale [124,125,126], acoustic imaging in thin films and microstructures on opaque substrates [123,124,127,128,129], material properties measurement of thin film [130], measurement of switching activity (changes of signal values) [131], and non-destructive evaluation of micrometric diamond films [120]. The duration of the laser pulse can be tuned to produce acoustic wavelength as short as 5 nm and provide volumetric information about the structure they travel in [123,124]. Ultrafast optical laser ultrasonics can generate and detect gigahertz–terahertz ultrasonic waves by using ultrashort light pulses and very high repetition rates for the excitation pulse. This method can be adopted to material characterizations, non-destructive evaluation and etc. that require nanometer spatial resolution.

**Figure 19:**
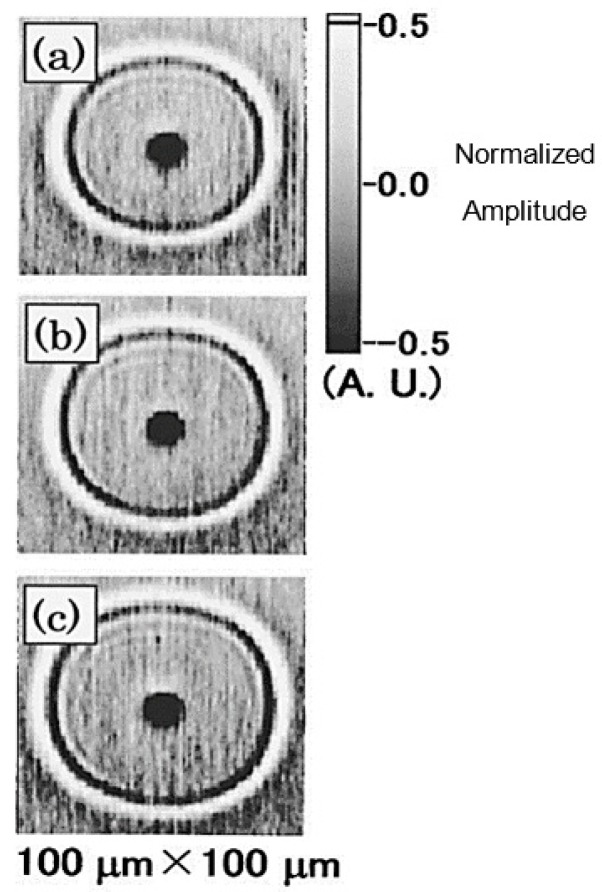
Experimental images of surface acoustic wave fronts for a Si substrate coated with a 50 nm gold film taken with 100 × 100 pixels at delay times, (**a**) 7.9, (**b**) 8.5, and (**c**) 9.1 ns [127]. These images took around 4 min to be produced.

Real-time imaging/signal processing can shorten the inspection time of the IC packaging under evaluation. In case of optical pump-probe lasers, real-time imaging of the ultrasonic waves generated by optical ultrafast lasers have been reported in the literature (Figure 19) and the current time of producing image for ultrasonic waves including, data acquisition, and analysis can be vary from 4 min up to 22 min depending on number of pixels in the image [127,132]. Summary of the NDT methods for IC packaging are detailed and presented in Table 1 for comparison purposes.

## 4. Discussion and Recommendations

The crucial requirements for an in-line inspection system can be listed as proper data acquisition, accurate data processing, suitable user interface and flexibility of the operation. To improve the current in-line systems, solutions such as utilizing high speed detectors, intensity control (for X-ray to control the image quality as an instance) and real-time image processing allows quick and accurate defect detection. In order to inspect hard-to-access areas, some advanced techniques like phased array can be used to improve the limited accessibility of the current NDT methods. Utilization of phased arrays techniques has a long history for NDT applications back in 1980s [133], and the technique has been adopted numerously since then [134]. In one of the most recent research studies, Tian et al. [135] presented a noncontact generic phased array beamforming technique for rapid defect inspection of anisotropic composite laminates. The technique provides an efficient solution that quickly identifies and locates defects even if there are located in hard to access areas. Utilization of phased array beamforming technique for the case of IC packaging inspection can be very helpful due to the following reasons: (1) Typically ultrasonic waves generated by noncontact lasers are very weak, but using a phased array and the concept of time reversal, ultrasonic waves can be focused at a specific point of interest even when the target inspection point is located in hard-to-access areas without moving the specimen, (2) Using a multi-channel phased array rather than a single channel probe, the imaging process time can be significantly reduced to improve signal-to-noise-ratio and, (3) volumetric inspections is possible due to the ability to focus at multiple depths.

**Figure 20:**
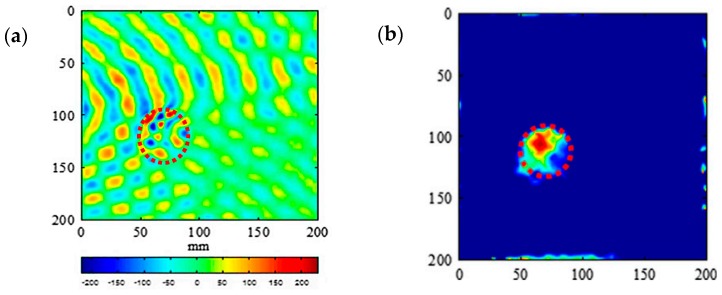
Comparisons of two snapshots of a composite plate with delamination defect under inspection with guided wave method (marked with a dashed red circle); (**a**) time domain out-of-plane displacement response of a composite plate and (**b**) frequency-wavenumber visualization of defect [141].

Advanced signal processing techniques is essential to extract features, which are not easily discernable from the measurements, and the signal processing techniques drastically enhance the capabilities of the NDT methods. The signal processing techniques should be robust to noise and able to remove unwanted noises from the measured data. A number of advanced signal processing techniques such as frequency-wavenumber (f-k) filtering [136,137,138,139,140], have been developed to evaluate the response spectrum and effectively extract standing wave components to assess the presence of defect (Figure 20). 

For feature extractions, Bayesian Networks (BN) has been utilized successfully to solve a diverse group of classification problems with regards to NDT applications [67]. ANN also provides powerful pattern classification and recognition capabilities and have been widely adopted for IC packaging inspection methods [52]. Deeper insight into more advanced signal processing for inspection and non-destructive applications in different stages such as data acquisition, feature extraction and diagnosis and prognosis can be found in Yn et al. [142].

In current microelectronic packaging solutions, AOI and AXI are very common to inspect both the interior and exterior microelectronic packaging, but the accuracy, resolution and speed of the methods are still under development. To address the aforementioned challenges regarding 3D microelectronic packages in Section 2, the best probable solution is most likely to combine methods such as IRT and 3D X-ray linked with proper modelling to overcome the shortcomings of each method. Future directions of NDT methods may include: (1) development of hybrid methods for defect detection such as combining promising methods like lock-in thermography and 3D CT or optical and infrared imaging, (2) more improvements and development in hybrid analysis techniques such as ANN and genetic algorithm.

## 5. Conclusions

In this paper, a review on the past and current non-destructive testing methods for IC package inspection is provided with the merits and limitations of each method. In the review, some of the most representative research studies and significant achievements have been addressed and commented upon. Furthermore, advanced candidate technologies for future IC package inspection and recommendations for improvement of the NDT methods have been recognized and discussed. The following summary observations are offered, together with some challenging issues for future development:

- For all the inspection methods with sophisticated signal processing and interpretation software, reducing the processing time of assessment and interpretation of signals and images are still challenging. These issues should be addressed for the development of an effective in-line inspection system.
- Noncontact methods seem to be more attractive due to the potential for in-line inspection, and less preparation time for inspection.
- The best probable solution to overcome the current challenges is to utilize hybrid methods linked to proper modelling.
- As the dimensions of the IC packaging (voxel size) continue to decrease, the micron range spatial resolution of the current NDT methods should be improved to submicron and nanometer ranges.
- To improve the current NDT methods, the followings can be considered; combination of numerical modeling with the NDT method such as X-ray and SAM, utilization of advanced signal processing (such as f-k filtering) and a phased array technique to access hard-to access areas.
- The ultrafast optical lasers offer the following unique characteristics for the inspection of IC packaging such as; the nanometer resolution, tunable wavelength of the ultrasonic waves depending on the target defect sizes, reasonable inspection time (currently it is in the order of couple of minutes) and being noncontact. While further investigation is warranted to discover more about the challenges, it is an objective of the present review to promote consideration and application of ultrafast optical lasers in the field.

## Figures and Tables

**Table 1:** Summary of the NDT methods for IC packaging.

NDT Method	Principle	Advantages and Type of Defect Can Detect	Limitations	Comment
**Scanning Acoustic Microscopy (SAM)**	A transducer generates the ultrasonic signal at specific frequency. The signal can propagate through the specimen, and it reflects back when there are discontinues or disturbance from the material.	Noncontact; Promising to detect delamination, large voids, non-uniform underfill and crack.	Poor resolution in µm and sub µm; Not suitable for in-line inspection application; Trade-off between resolution and penetration depth; Poor sensitivity to cracks; Requirement for coupling medium; Poor sensitivity to edge defects.	Resolution can be increased by going to higher frequencies (GHz), but then the penetration depth reduces. Trade-off between resolution and penetration depth.
**Surface Acoustic Waves (SAW)**	Laser/ PZT induced ultrasonic waves for excitation and Doppler effect for noncontact optical vibration measurements.	Mostly noncontact; Promising to detect delamination, large voids and non-uniform underfill.	The accessibility of the electronic packaging is crucial; Less sensitive to in-depth defects; Poor resolution in sub µm level; More sensitive for thin chips; Reference data required to assess the presence of defect.	
**X-Ray**	Transmission of X-ray by a source through an object and a receiver receives the transmitted energy. From the transmitted energy the internal condition can be assessed based on the defined characteristics of the healthy and faulty states.	Promising to detect delamination, large voids cracks and misalignments. Can be combined with other methods; 3D CT proved to be successful for 3D packaging; In-line application (AXI); Suitable for both inner and outer inspections; Significant improvement to answer the industry needs over the past decade.	Conventional methods were destructive; Poor resolution in sub µm level; Long processing time (in the order of hours).	The methods associated with X-ray, are the most common methods utilized in the inspection applications.
**Thermography**	To detect heat radiation of a body within the electromagnetic spectrum region by IR camera detector and produce images of the heat distribution. From the temperature variation of the recorded images the internal condition of the body can be assessed.	Noncontact; Promising for crack, missing solder bump, delamination and void; Promising to inspect 3D packaging (lock-in thermography).	Thermal noise can really affect the results; Spatial resolution for sub-micron levels. Signal difference in the defects in sub µm level is weak; Limited thickness of inspection under the surface; Overheating problem for some very sensitive packaging.	
**Infrared Microscopy (IRM)**	Infrared Microscopy (IRM) denotes a microscopy achieved at infrared wavelengths. Objective lenses and Illuminators are to facilitate magnifications and bright field imaging.	Noncontact; Promising to detect void and crack in solder bumps.	Suitable only for µm defect range; Cannot penetrate through metal or thick underfill.	
**Time Domain Reflectometry (TDR)**	Sending an electrical pulse and detecting reflections returning from impedance discontinuities along the controlled-impedance transmission path.	Noncontact; Ability to access hard-to access areas; Confirmed successful to detect short failures in 3D packaging; Promising for crack detection.	The application is limited only to conductive materials; Reference data required to assess the presence of defect	
**Magnetic Current Imaging (MCI)**	Measurements of the magnetic field associated with a flowing current and map out hidden current-carrying wires by measuring the magnetic fields around them. To locate the defect, the current density images are compared with defect–free samples.	Noncontact; Suitable for mostly short circuit faults.	Reference data required to assess the presence of defect; Poor resolution for sub-micron order.	
**Ultrafast optical lasers (femtosecond/picosecond)**	Based on generation of ultrashort pulse lasers on extremely short time intervals. Once a pump pulse hits on the surface of an opaque solid, it produces strain pulse. From the reflection sensing the internal condition of the material can be inspected or characterized.	Noncontact; Suitable resolution in sub µm and nm; The wavelength of the excited waves can be tuned to the suitable size.	Large data size, and complex post processing; The other limitations are yet to be discovered for the inspection of the IC packaging.	
